# Editorial: Rediscovering local landraces: shaping horticulture for the future, volume II

**DOI:** 10.3389/fpls.2023.1329995

**Published:** 2023-12-14

**Authors:** Spyridon A. Petropoulos, Lillian Barros, Isabel C.F.R. Ferreira

**Affiliations:** ^1^ Laboratory of Vegetable Production, Department of Agriculture, Crop Production and Rural Environment, University of Thessaly, N. Ionia, Greece; ^2^ Centro de Investigação de Montanha (CIMO), Instituto Politécnico de Bragança, Bragança, Portugal; ^3^ Laboratório Associado para a Sustentabilidade e Tecnologia em Regiões de Montanha (SusTEC), Instituto Politécnico de Bragança, Bragança, Portugal

**Keywords:** tomato germplasm, *Allium sativum*, yum, mungbean, genotypic diversity, squash, drought stress

The ongoing climate change accompanied by weather extremes has increased uncertainty in the crop production sector, with a severe impact on yield and quality of crops (FAO, 2022). Moreover, the increasing world population, along with the continuous reduction of available irrigation water and agricultural land degradation due to anthropogenic activities, necessitates the redesign of the existing farming systems through the integration of valuable and underexplored genetic material, such as the local landraces of various vegetable species. Local landraces are cultivated in restricted regions and have been adapted over the years to specific growing conditions (soil and climate characteristics). Usually, they possess high genotypic diversity, which allows the crops to overcome the pressure from abiotic and biotic stressors from time to time (Conesa et al., 2020), while increasing on-farm agrobiodiversity at the same time (Conversa et al., 2020). For this reason, this genetic material is highly valuable for breeding purposes and the selection of new genotypes with improved characteristics (Formisano et al., 2012). Their use is becoming more and more limited due to the intensification of crop production sections and restrictions from marketing standards mostly related to visual appearance and the uniformity of the final product. However, the current trends show increasing interest not only from farmers who seek alternative farming options in the climate change scenario but also from consumers who seek products of known origin and high quality that have been produced in a sustainable manner.

The present Research Topic is a follow-up to the first volume of “*Rediscovering Local Landraces: Shaping Horticulture for the Future*” (Petropoulos et al., 2019) and brings together several research articles and review reports. It focuses on highlighting the necessity to preserve local landraces and integrate them into sustainable horticultural crop production systems while emphasizing the special features of this genetic material.

In the work of Farinon et al., research was conducted on the phenotypic and genotypic diversity of Italian tomato germplasm collected in the Lazio region. For this purpose, the authors evaluated 32 accessions, including eight locally specified landraces and 19 accessions with no particular name. The results of this study showed a high phenotypic diversity for many attributes related to plant morphology, fruit quality, and yield parameters. Multivariate analysis identified two distinct groups that differed in fruit shape, while genotyping analysis using SNPs identified accessions that could be derived from modern genetic material and commercially available cultivars. The study by Tagiakas et al. was also related to the characterization and evaluation of various Greek tomato landraces based on yield and quality parameters, with the aim of identifying genotypes that could be integrated into sustainable, low-input farming systems. The study included 22 local landraces and two commercially available genotypes, which were characterized via phenotypic markers. The experiment was conducted in two phases, where in Phase 1, the most promising landraces in terms of fruit quality and yield were selected and further evaluated under low-input conditions using the intrapopulation “pure line selection” method for two consecutive years. The obtained results provided important information regarding the quality of the landraces studied while allowing the identification of the most promising genotypes that could be further valorized in low-input cultivation systems. Casals et al. investigated the phenotypic diversity and distinct qualitative characteristics of the Belltall garlic landrace, which is commonly cultivated in Catalonia, Spain. The results of this study showed that most of the agromorphological traits were genetically controlled. However, plants grown outside this specific region lost qualitative features related to the appearance of the clove (e.g., color and size), suggesting a genotype × environment interaction and local adaptation for these particular attributes.

Han et al. focused on mapping the genotypic variation in mungbean through the resequencing of 558 Chinese landraces and their phenotyping in six different locations. The results of this study provided a new insight into the genetic structure of mungbean through the identification of several signals (110) that were associated with nine agronomic traits controlled by different genes, which could be further exploited in breeding programs.

Agre et al. studied the yam landraces of Nigeria and proceeded to assess their genetic variability patterns, qualitative trait loci, alleles, and genetic characteristics. Their analysis revealed three distinct groups, based on thirteen SNP markers correlated with specific agronomic traits. Moreover, the authors identified thirteen landraces with high crossing merit for several traits through the use of Genomic Prediction of Cross Performance, which could be used as genitors in yam breeding programs.

According to Saadaoui et al., squash is a species with high genotypic variability, mostly due to interspecific hybridization, which could be exploited in the selection of elite genotypes adapted to drought stress. In their study, they evaluated four Tunisian squash landraces in terms of tolerance to water stress at two distinct growth phases, namely seed germination and the young seedling stage. Their results highlighted the drought tolerance of two specific landraces (Batati orange and Bejaoui green), as indicated by higher seed germination rates and seedling growth at the tested drought stress levels ((-0.24, -0.47, and -0.73 MPa).

Finally, Rivas et al. conducted a literature review on the genetic variability of vegetable and maize landraces in southern Brazil and the Uruguayan Pampas, with the aim of compiling all the relevant information that could be used in conservation and valorization strategies aimed at sustainable management of the existing genetic material. They also presented case studies of *in situ* and *ex situ* conservation of several landraces to identify problems and highlight the genetic variability of the species in these regions.

In summary, this Research Topic comprises seven studies (six research articles and one review) that provide significant knowledge on local landraces of vegetables in different regions of the world. The presented information could be useful in highlighting the importance of the genetic variability captured in local landraces, which could be used in valorizing the existing genetic material through breeding efforts to address the pressure of climate change on crops.

## Author contributions

SP: Writing – original draft, Writing – review & editing. LB: Writing – original draft, Writing – review & editing. IF: Writing – original draft, Writing – review & editing.
